# CYP4F18-Deficient Neutrophils Exhibit Increased Chemotaxis to Complement Component C5a

**DOI:** 10.1155/2015/250456

**Published:** 2015-11-03

**Authors:** Rachel Vaivoda, Christine Vaine, Cassandra Boerstler, Kristy Galloway, Peter Christmas

**Affiliations:** ^1^Nephrology Division, Department of Medicine, Massachusetts General Hospital, Charlestown, MA 02129, USA; ^2^Department of Biology, Radford University, Radford, VA 24142, USA

## Abstract

CYP4Fs were first identified as enzymes that catalyze hydroxylation of leukotriene B_4_ (LTB_4_). CYP4F18 has an unusual expression in neutrophils and was predicted to play a role in regulating LTB_4_-dependent inflammation. We compared chemotaxis of wild-type and *Cyp4f18* knockout neutrophils using an *in vitro* assay. There was no significant difference in the chemotactic response to LTB_4_, but the response to complement component C5a increased 1.9–2.25-fold in knockout cells compared to wild-type (*P* < 0.01). This increase was still observed when neutrophils were treated with inhibitors of eicosanoid synthesis. There were no changes in expression of other CYP4 enzymes in knockout neutrophils that might compensate for loss of CYP4F18 or lead to differences in activity. A mouse model of dextran sodium sulfate colitis was used to investigate the consequences of increased C5a-dependent chemotaxis *in vivo*, but there was no significant difference in weight loss, disease activity, or colonic tissue myeloperoxidase between wild-type and *Cyp4f18* knockout mice. This study demonstrates the limitations of inferring CYP4F function based on an ability to use LTB_4_ as a substrate, points to expanding roles for CYP4F enzymes in immune regulation, and underscores the *in vivo* challenges of CYP knockout studies.

## 1. Introduction

CYP4Fs are a family of cytochrome P450 (CYP) enzymes that were first identified for their ability to catalyze end-chain hydroxylation and inactivation of leukotriene B_4_ (LTB_4_) [1]. The potency of LTB_4_ as an inflammatory mediator in normal immune responses and pathologies is well established. LTB_4_ is generated by the 5-lipoxygenase pathway of arachidonic acid metabolism and is implicated in the progression of diverse immune disorders such as inflammatory bowel disease, ischemia-reperfusion injury (IRI), arthritis, and asthma [2, 3]. Therefore, CYP4Fs are predicted to play a significant role in the regulation of inflammation and prevention of disease. There is growing evidence to support this possibility. CYP-dependent LTB_4_ hydroxylase activity limits neuroinflammation in mouse models [4] and might contribute to the beneficial effects of retinoids in the treatment of inflammatory skin diseases [5, 6]. Neutrophils and colonic mucosa from patients with inflammatory bowel disease have reduced LTB_4_ hydroxylase activity [7, 8], and genetic association studies link variants of the* CYP4F2* and* CYP4F3* genes with celiac disease and Crohn's disease [9, 10].

Human neutrophils have been used for detailed studies of CYP-dependent LTB_4_ metabolism: hydroxylation at the terminal (*ω*) position generates 20-hydroxy LTB_4_, which is subsequently converted to the inactive metabolite 20-carboxy LTB_4_ [11]. This is the major pathway for the inactivation of LTB_4_ in human neutrophils [12, 13]. The enzyme responsible for the initial *ω*-hydroxylation step was identified as CYP4F3 [14, 15], and subsequently we demonstrated that this enzyme is an alternative splice form of the* CYP4F3* gene designated as CYP4F3A [16]. A second splice form, CYP4F3B, has lower activity for LTB_4_ and is expressed in different locations such as liver and kidney [17]. The unusual localization and high expression of CYP4F3A in human neutrophils, and its high activity for LTB_4_ as a substrate, suggest that inactivation of LTB_4_ is a specialized function of the enzyme. There is evidence for temporal expression of CYP4Fs consistent with the resolution phase of inflammation in some experimental models [18], but expression of CYP4F3A in neutrophils does not fit this time frame. Neutrophils are short-lived cells associated with the early stages of inflammation, and CYP4F3A is expressed at a high constitutive level both before and during inflammatory recruitment of the cells [19]. It is possible that LTB_4_ inactivation functions to restrain neutrophil infiltration and prevent excessive inflammation. An alternative possibility is that LTB_4_ inactivation plays a role in neutrophil polarization, which is required to maintain normal chemotaxis [20].

We developed mouse models to better understand the role of CYP4Fs in neutrophil-dependent inflammation. We identified the CYP4F18 enzyme as the mouse homologue of CYP4F3A [21] and generated targeted deletions in the* Cyp4f18* gene. Neutrophils from* Cyp4f18* knockout mice exhibit a null phenotype for end-chain hydroxylation of LTB_4_ [22]. However, there are significant differences between mice and humans. The* Cyp4f18* gene is not alternatively spliced and generates a single enzyme that is homologous to CYP4F3A in sequence, localization to neutrophils, and high activity for LTB_4_. The products of end-chain hydroxylation by CYP4F18 are 19-hydroxy LTB_4_, and to a lesser extent 18-hydroxy LTB_4_, not 20-hydroxy LTB_4_ [21, 22]. It is not known whether *ω*-1 and *ω*-2 hydroxylation of LTB_4_ represents an efficient inactivation pathway. Furthermore, mouse neutrophils have an alternative pathway of LTB_4_ metabolism that involves a 12-hydroxydehydrogenase. Knockout of* Cyp4f18* does not impact neutrophil infiltration into kidney tissue and disease pathology in a mouse model of renal IRI [22], although inhibition of LTB_4_ synthesis does have observable effects in this model [23]. It appears that CYP4F18 is redundant for LTB_4_ inactivation in mouse neutrophils, and we speculated that it might have an alternative function in these cells.

Since the discovery of CYP4Fs, numerous* in vitro* substrates have been identified [1]. There are 7 members of the human CYP4F family including the two splice forms of CYP4F3 (4F2, 4F3A, 4F3B, 4F8, 4F11, 4F12, and 4F22) and 9 members of the mouse family (4F13, 4F14, 4F15, 4F16, 4F17, 4F18, 4F37, 4F39, and 4F40). CYPs typically have broad and overlapping substrate specificity, and a single enzyme such as CYP4F3B might have the capacity to catalyze multiple reactions including inactivation of LTB_4_, generation of 20-hydroxyeicosatetraenoic acid (20-HETE), and modification of fatty acid epoxides [1, 24]. This suggests potentially diverse and prominent roles for CYP4Fs in immune regulation but creates a challenge for the identification of physiologically relevant substrates [25]. It is possible that CYP4Fs have different functions in different tissue locations, and new experimental systems will be required to determine the significance of particular reactions and disentangle the effects of multiple CYP4Fs. CYP4F18 is the only CYP4F family member expressed at high levels in mouse neutrophils, so* Cyp4f18* knockout mice provide a novel system to dissect diversity of function. In this report we demonstrate that neutrophils from* Cyp4f18* knockout mice show increased chemotaxis to complement component C5a that is independent of LTB_4_, an unexpected result that is not predicted by known CYP4F substrates.

## 2. Materials and Methods

### 2.1. Mice

*Cyp4f18* knockout (−/−) mice were generated in a C57BL/6 background as previously reported [22] and are available at the Mutant Mouse Regional Resource Center (MMRRC) with the designation B6.129S4(Cg)-Cyp4f18^tm1.1Pchr^.* Cyp4f18* +/− heterozygous mice were maintained at the Massachusetts General Hospital and mated to generate* Cyp4f18* −/− homozygous knockouts and* Cyp4f18* +/+ wild-type littermates for experiments. Genotyping assays are as previously described [22]. Breeding and experimentation of mice were performed in accordance with the guidelines of the Massachusetts General Hospital/Partners Committee on Research Animal Care. Mice of 6–12 weeks of age were used for experiments.

### 2.2. Isolation of Bone Marrow Neutrophils

Mouse bone marrow cells were isolated from femurs and tibias by perfusion with phosphate buffered saline (PBS). The cells were filtered through a 40 *μ*m cell strainer, washed in PBS, and layered on top of a discontinuous two-layer gradient of Histopaque 1077/1119 (Sigma). After centrifugation at 700 ×g for 30 min at RT, neutrophils were separated from other cells including erythrocytes and recovered at the interface of the 1077 and 1119 fractions. Purity of the isolated polymorphonuclear cells was confirmed (>90%) by staining nuclei with DAPI and examination under a Nikon Eclipse Ti microscope with confocal imaging. Viability of the cells (>95%) was assessed by trypan blue exclusion.

### 2.3. *In Vitro* Chemotaxis Assay

Bone marrow neutrophils were isolated as described above and resuspended in migration medium (RPMI + 0.5% FBS) to a concentration of 3 × 10^6^ cells/mL. Chemotaxis was performed using a 12-well plate with Transwell 12 mm polycarbonate membrane inserts of 3.0 *μ*M pore size (Corning 3402). The cells were placed in the insert (0.5 mL, 1.5 × 10^6^ cells per insert), and 1.5 mL of migration medium containing different concentrations of chemoattractant was placed in the lower well. Migration medium containing vehicle but no chemoattractant was used as a control to measure background chemotaxis, and chemoattractant was added to both the insert and the lower well as a control for chemokinesis. The 12-well plates were incubated for 3 hours at 37°C in a 5% CO_2_ incubator. The medium in the lower well was transferred to a 12 × 75 mm sterile culture tube (BD Falcon), and the well was washed with PBS. The tubes were then centrifuged for 5 min at 200 ×g, and the cell pellet was resuspended in 0.1 mL migration medium. The total number of cells was determined using a hemocytometer. To calculate the chemotactic index, the number of cells migrated in response to chemoattractant was divided by the number of spontaneously migrated cells (background).

The chemoattractants tested were LTB_4_ (Cayman), mouse complement component C5a (R&D Systems), mouse CXCL1/KC (R&D Systems), and WKYMVdM peptide (Sigma) as an agonist for the mouse formyl peptide receptor. In some experiments, bone marrow neutrophils were incubated with or without inhibitors of eicosanoid synthesis or BLT1 for 30 min at 37°C prior to adding the cells to the 12-well plate inserts. This included incubations with 0.5 *μ*M of the FLAP inhibitor MK 886 (Cayman 10133), 1 *μ*M of a cPLA2*α* inhibitor (Calbiochem 525143, PubChem CID 9833099), and 10 *μ*M of the BLT1 antagonist LY223982 (Cayman 10010024). Each experimental condition was performed in duplicate or triplicate on a single 12-well plate, and each experiment was performed at least 4 times (*n* ≥ 4).

### 2.4. Flow Cytometry

Bone marrow cell suspensions in PBS + 0.5% BSA were preincubated with Mouse Fc Block (BD Biosciences) for 5 min at 4°C (0.5 *μ*g/10^6^ cells/100 *μ*L) and then incubated with fluorophore-conjugated anti-mouse monoclonal antibodies for 30 min at 4°C (antibodies diluted to 1 *μ*g/mL). Anti-CD45-PerCP (clone 30-F11) and anti-Ly6G-FITC (clone 1A8) were from BD Biosciences. Anti-C5aR(CD88)-APC (clone 20/70) was from BioLegend. The cells were fixed in BD stabilizing fixative (BD Biosciences). Labeled cells were analyzed at the Flow Cytometry Core Facility, Massachusetts General Hospital, using a BD SORP 7 Laser LSRII and FlowJo software, as in previous studies [19, 22]. The cells were gated on forward versus side scatter, then for CD45 expression, prior to double plot analysis of Ly6G and C5aR (numbers were assigned to each quadrant to indicate percentage of total CD45+ cells). Bone marrow samples from 5 wild-type mice and 5* Cyp4f18* knockout mice were analyzed.

### 2.5. RNA Isolation and Real Time PCR

Total RNA was isolated from cells and tissues using the RNeasy Plus Mini Kit with QIAshredder (Qiagen). Reverse transcription was performed with a High Capacity cDNA Reverse Transcription Kit (Life Technologies, Applied Biosystems). The cDNA was analyzed for target gene expression using TaqMan primer sets and a StepOnePlus real time PCR machine from Applied Biosystems. A standard reaction protocol was followed (50°C for 2 min, 95°C for 10 min, 40 cycles of 95°C for 15 sec and 60°C for 1 min). Relative quantification of gene expression in knockout samples compared to wild-type was performed by the ΔΔCt method using mouse GAPDH as endogenous control. Each sample was run in triplicate to determine ΔCt values, 2^−ΔCt^ values, or fold-differences in expression (2^−ΔΔCt^). Values from four experiments were expressed as mean ± SEM. The TaqMan primer-probe sets were purchased from Life Technologies (Applied Biosystems) as listed in Table 1.

### 2.6. Dextran Sodium Sulfate (DSS) Colitis

DSS (MP Biomedicals) of average molecular weight 42 kDa (35–50,000) was administered to 8-week-old mice ad libitum at a concentration of 4% in drinking water for 9 days. Control mice received the same drinking water without DSS (*n* = 10 mice in each group). Changes in body weight were calculated every day. A disease activity index (DAI) was determined by assigning a score of 1–4 for weight loss (1: 1–5%, 2: 5–10%, 3: 10–15%, and 4: >15%), rectal bleeding (ranging from 1: positive to 4: gross bleeding), stool consistency (ranging from 1: loose stools to 4: diarrhea with fecal material adherent to anal fur), and body posture/lethargy (ranging from 1: mild to 4: severe). The total scores were averaged to give a value between 0 (normal) and 4 (maximum). Mice were sacrificed on day 9, and colonic tissue samples were collected for further analysis. Myeloperoxidase (MPO) was measured using an ELISA kit from Hycult Biotech (HK210). The assay was performed on tissue samples that had been frozen and stored at −70°C. The samples were thawed, weighed, and homogenized in a lysis buffer containing 200 mM NaCl, 5 mM EDTA, 10 mM tris, 10% glycerin, 1 mM PMSF, 1 *μ*g/mL leupeptin, and 28 *μ*g/mL aprotinin, pH 7.4 (20 *μ*L lysis buffer per mg tissue). The homogenate was transferred to a 1.5 mL microfuge tube and centrifuged two times at 1500 ×g for 15 min at 4°C. Supernatants were snap frozen in liquid nitrogen and stored at −70°C. MPO was assayed by ELISA according to the manufacturer's instructions.

### 2.7. Statistical Analysis

The data was analyzed using Graphpad Prism version 5 statistical software. The results are expressed as the mean ± SEM. A *t*-test was used for comparisons of paired data, and multigroup data were analyzed by ANOVA. A *P* value of less than 0.05 was considered significant.

## 3. Results

### 3.1. LTB_4_-Dependent Neutrophil Chemotaxis

**Figure 1:**
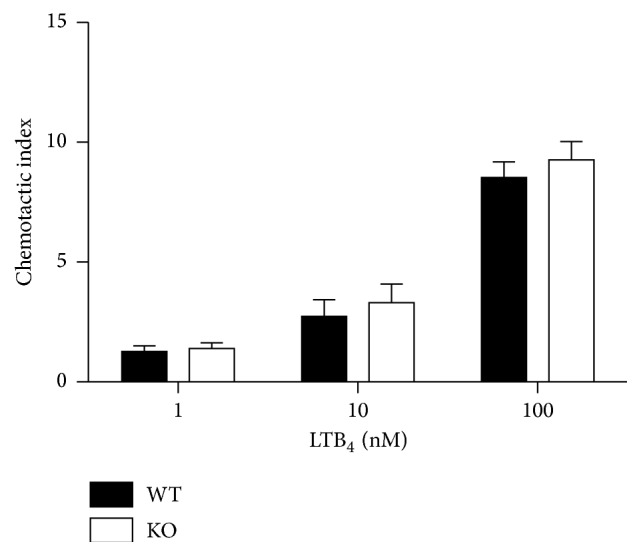
*Cyp4f18* knockout neutrophils show no change in LTB_4_-dependent chemotaxis compared to wild-type. Chemotaxis of bone marrow neutrophils from wild-type (WT) and* Cyp4f18* knockout (KO) mice was measured using LTB_4_ as a chemoattractant. The chemotactic index represents the number of cells migrated in response to LTB_4_ divided by background (error bars represent SEM, *n* = 10).

LTB_4_-dependent chemotaxis of neutrophils from wild-type and* Cyp4f18* knockout C57BL/6 mice was compared using an* in vitro* assay. A dose-dependent increase in chemotaxis was observed (Figure 1). For wild-type (WT) neutrophils, the chemotactic index increased from 1.27 ± 0.12 at 1 nM LTB_4_ to 2.73 ± 0.7 at 10 nM LTB_4_ and 8.53 ± 0.64 at 100 nM LTB_4_. For* Cyp4f18* knockout (KO) neutrophils, the chemotactic index increased from 1.4 ± 0.1 at 1 nM LTB_4_ to 3.3 ± 0.77 at 10 nM LTB_4_ and 9.27 ± 0.77 at 100 nM LTB_4_. At each concentration, there was no significant difference between the chemotactic index for wild-type and* Cyp4f18* knockout neutrophils (*n* = 10, *P* > 0.05).

### 3.2. C5a-Dependent Neutrophil Chemotaxis

**Figure 2:**
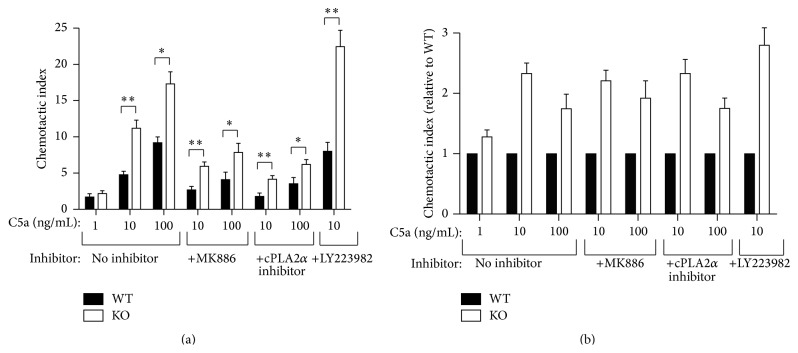
*Cyp4f18* knockout neutrophils show increased C5a-dependent chemotaxis compared to wild-type. Chemotaxis of bone marrow neutrophils from wild-type (WT) and* Cyp4f18* knockout (KO) mice was measured using C5a as a chemoattractant. Prior to chemotaxis, the neutrophils were incubated with or without the FLAP inhibitor MK 886 (0.5 *μ*M), a cPLA2*α* inhibitor (CID 9833099, 1 *μ*M), or a BLT1 antagonist (LY223982, 10 *μ*M). (a) Chemotactic index represents the number of cells migrated in response to C5a divided by background (error bars represent SEM, *n* = 5, ^*∗*^ *P* < 0.05, ^*∗∗*^ *P* < 0.01). (b) Schematic plot for the relative difference in chemotactic index in* Cyp4f18 *knockout neutrophils compared to wild-type for each experimental condition.

The complement component C5a was originally used as a control in these experiments, because C5a-dependent chemotaxis was not expected to be affected by loss of* Cyp4f18*. Surprisingly, significant differences in chemotaxis were observed when comparing wild-type and knockout neutrophils (Figure 2). For wild-type neutrophils, the chemotactic index increased from 1.7 ± 0.46 at 1 ng/mL C5a to 4.79 ± 0.48 at 10 ng/mL C5a and 9.2 ± 0.79 at 100 ng/mL C5a. For* Cyp4f18* knockout (KO) neutrophils, the chemotactic index increased from 2.2 ± 0.38 at 1 ng/mL C5a to 11.2 ± 1.1 at 10 ng/mL C5a and 17.3 ± 1.7 at 100 ng/mL C5a. There was a significant difference between the chemotactic index for wild-type and* Cyp4f18* knockout neutrophils at 10 ng/mL C5a (*P* < 0.01) and 100 ng/mL C5a (*P* < 0.05), but not at 1 ng/mL C5a (Figure 2(a)). Overall, the relative difference in chemotactic index for knockout compared to wild-type was 2.3-fold at 10 ng/mL C5a and 1.9-fold at 100 ng/mL C5a (Figure 2(b)). No significant differences between* Cyp4f18* knockout and wild-type neutrophil chemotaxis were observed when CXCL1/KC or WKYMVdM peptide was used as chemoattractant (data not shown).

Previous studies demonstrated that LTB_4_ is a signal relay molecule during neutrophil chemotaxis: LTB_4_ synthesis and secretion are induced by primary chemoattractants such as C5a, and this amplifies neutrophil migration [20, 26]. The amplification is reduced by MK 886 [20], a FLAP inhibitor that is known to block 5-lipoxygenase activation and leukotriene synthesis [27]. As expected, we observed a decrease in C5a-dependent chemotaxis in neutrophils treated with MK 886 (Figure 2(a)), but the decrease was equivalent in wild-type and* Cyp4f18* knockout neutrophils (0.45–0.56-fold), such that the relative difference in chemotactic index remained unchanged: 2.21-fold higher in* Cyp4f18* knockout neutrophils compared to wild-type at 10 ng/mL C5a and 1.92-fold higher at 100 ng/mL C5a (Figure 2(b)). Similar results were obtained when neutrophils were treated with a cPLA2*α* inhibitor: there was an equivalent decrease in C5a-dependent chemotaxis in wild-type and* Cyp4f18* knockout neutrophils of 0.3–0.37-fold (Figure 2(a)), and the relative difference in chemotactic index remained unchanged: 2.3-fold higher in* Cyp4f18* knockout neutrophils at 10 ng/mL C5a and 1.75-fold higher at 100 ng/mL C5a (Figure 2(b)). The cPLA2*α* inhibitor is less specific than the FLAP inhibitor: it blocks eicosanoid production by preventing release of arachidonic acid from membrane phospholipids [28] and therefore inhibits synthesis of a wide range of lipid mediators in addition to leukotrienes. Overall, the data show that loss of CYP4F18 results in increased neutrophil chemotaxis to C5a and suggest that this is independent of LTB_4_ and other eicosanoids.

LY223982 is a synthetic BLT1 (LTB_4_ receptor) antagonist that has been used to study neutrophil function [29]. We incubated neutrophils with LY223982 prior to chemotaxis assays using 10 ng/mL C5a. This resulted in a 1.7-fold increase in C5a-dependent chemotaxis in wild-type neutrophils and a 2-fold increase in* Cyp4f18* knockout neutrophils, compared to cells that were not treated with LY223982 (Figure 2(a)). There is previous evidence for cross-desensitization between neutrophil chemoattractant receptors [30], and this might account for our data: less cross-desensitization of the C5a receptor following inhibition of BLT1 could result in an increased chemotactic response to C5a. However this does not account for the differences between* Cyp4f18* knockout and wild-type neutrophils, because the relative difference in chemotactic index remained undiminished following treatment with LY223982: 2.8-fold higher in* Cyp4f18* knockout neutrophils compared to wild-type (Figure 2(b)).

### 3.3. Comparison of Gene Expression in Cyp4f18 Knockout and Wild-Type Neutrophils

**Figure 3:**
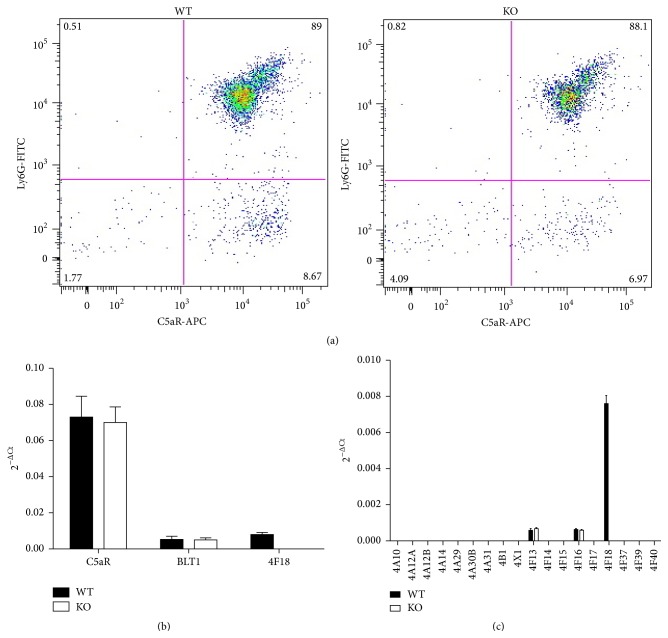
Gene expression in wild-type and* Cyp4f18* knockout neutrophils. Flow cytometry analysis of bone marrow cells from wild-type (WT) and* Cyp4f18* knockout (KO) mice using antibodies to Ly6G (neutrophil marker) and C5aR; representative double plots from individual mice are shown (a). Reverse transcription and real time PCR analysis of C5aR mRNA expression (b) and CYP4 mRNA expression (c), in isolated bone marrow neutrophils. ΔCt values were determined for each transcript using GAPDH as endogenous control, and 2^−ΔCt^ values were plotted for WT and KO samples (*n* = 4). Relative quantitation (2^−ΔΔCt^) determined that there was no significant change in expression of any of the transcripts tested in knockout samples compared to wild-type, except for the loss of CYP4F18.

We have previously used multicolor flow cytometry analysis of cells from mouse bone marrow and other tissues to investigate protein expression in different cell lineages [19, 22]. Ly6G is a component of the myeloid marker Gr-1 (ly6G + Ly6C) that is preferentially expressed in neutrophils, and fluorophore-conjugated monoclonal antibodies to Ly6G are useful to identify neutrophils in flow cytometry studies [22]. Using this approach, mouse bone marrow cells were stained with anti-Ly6G-FITC and anti-C5aR-APC, and analysis of double plots shows an equivalent level of C5a receptor (C5aR) expression in neutrophils from* Cyp4f18* knockout and wild-type mice (Figure 3(a)). Real time PCR analysis of isolated bone marrow neutrophils (Figure 3(b)) determined that there is no significant difference in mRNA expression of C5aR or BLT1 in* Cyp4f18* knockout neutrophils compared to wild-type (*n* = 4, *P* > 0.05). A plot of 2^−ΔCt^ values shows that C5aR is expressed at approximately 10-fold higher levels than BLT1 in the cells (Figure 3(b)). The values for relative expression (2^−ΔΔCt^) of C5aR in knockout neutrophils compared to wild-type ranged from 0.92 to 1.05.

A real time PCR assay designed to detect exons 8 and 9 of Cyp4f18 was used to confirm loss of expression of these exons in knockout mice, as previously described [22]. We routinely performed real time PCR analysis of isolated bone marrow neutrophils to determine if changes in expression of other* Cyp* genes might account for differences in the* Cyp4f18* knockout. We have previously shown that* Cyp4f18* is the predominant* Cyp4f* subfamily member in bone marrow neutrophils and that* Cyp4f13* and* Cyp4f16 *are detected at lower levels [22]. In this study, we extended the analysis to include all* Cyp4* family members in mouse (Figure 3(c)). No other* Cyp4* transcripts were detected in wild-type or knockout neutrophils. Importantly, there were no changes in* Cyp4* expression in knockout neutrophils that might compensate for the loss of* Cyp4f18* or lead to differences in activity of the cells.

### 3.4. Mouse Model of DSS Colitis

**Figure 4:**
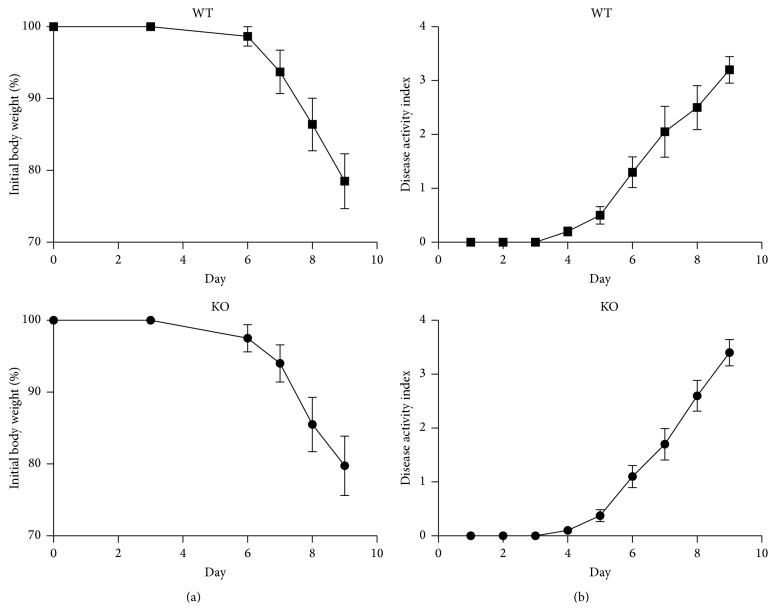
Mouse model of DSS colitis. Wild-type (WT) and* Cyp4f18* knockout (KO) mice were treated with 4% DSS in drinking water for 9 days, and the change in body weight (a) or disease activity index (b) was measured each day. There were no significant differences between WT and KO mice (data shows mean values ± SEM, *n* = 10, *P* > 0.05).

**Figure 5:**
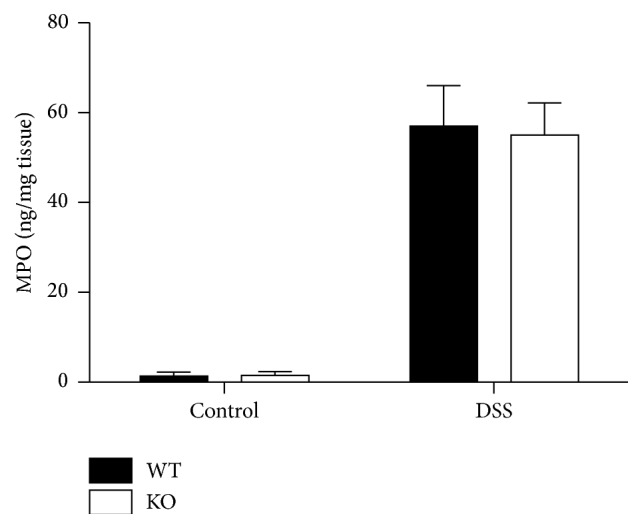
Myeloperoxidase (MPO) levels in colonic tissue in DSS colitis. MPO was measured by ELISA in colonic tissue homogenates from wild-type (WT) and Cyp4f18 knockout (KO) mice treated for 9 days with 4% DSS in drinking water (error bars represent SEM, *n* = 5). Control mice received drinking water without DSS.

A mouse model of DSS colitis was used to investigate the consequences of increased C5a-dependent chemotaxis* in vivo* (Figure 4). Inhibition of C5a activity has been shown to reduce disease pathology in this model in C57BL/6 mice [31]. Continuous administration of 4% DSS in drinking water to 8-week-old mice resulted in a rapid decline in body weight from 98.7 ± 1.35% of initial weight on day 6 to 78.5 ± 3.8% of initial weight on day 9 in wild-type mice. There was a corresponding increase in disease activity index (maximum = 4) from 1.3 ± 0.29 on day 6 to 3.2 ± 0.24 on day 9. There were no significant differences in the values for loss of weight or increase in disease activity in* Cyp4f18* knockout mice (*n* = 10, *P* > 0.05). MPO, an enzyme produced mainly by neutrophils, was measured in colonic tissue to quantify inflammatory cell infiltration (Figure 5). On day 9, the MPO level was 57 ± 9.0 ng/mg tissue in wild-type mice and 55 ± 7.1 ng/mg tissue in* Cyp4f18* knockout mice (*n* = 5, *P* > 0.05). Based on the similarity of these indicators, we did not proceed with further histological analysis of colon tissue.

## 4. Discussion

We previously generated* Cyp4f18* knockout mice [22], and predicted that the mice would exhibit altered LTB_4_-dependent inflammation based on the ability of the CYP4F18 enzyme to metabolize LTB_4_ in neutrophils. However, there were no significant differences in inflammation and injury in a mouse model of renal IRI compared to wild-type [22], although inhibition of LTB_4_ synthesis does ameliorate pathology in this model [23]. The results of an* in vitro* chemotaxis assay are consistent with these previous observations* in vivo*. LTB_4_ stimulated chemotaxis of neutrophils in a dose-dependent manner, but there were no significant differences in the response of* Cyp4f18* knockout and wild-type cells (Figure 1). In the renal IRI model, we measured infiltration of neutrophils into kidney tissue by traditional histological approaches and flow cytometry and demonstrated a comparable time course and magnitude of infiltration in* Cyp4f18* knockout and wild-type mice [22]. However, the loss of CYP4F18 might be compensated by other CYP4Fs in the complex tissue physiology of inflammation. The* in vitro* assay reported here confirms that the similarity between wild-type and knockout is inherent to neutrophils.

There are a number of possible explanations for the unaltered chemotactic response to LTB_4_ in* Cyp4f18* knockout neutrophils. CYP4F18 converts LTB_4_ to 19-hydroxy LTB_4_, and to a lesser extent 18-hydroxy LTB_4_, in mouse neutrophils, not 20-hydroxy LTB_4_ as seen in humans. These products were not detected in* Cyp4f18* knockout neutrophils [22], but it is not known whether *ω*-1 and *ω*-2 hydroxylation of LTB_4_ represents an efficient inactivation pathway. These studies underscore the importance of accurately identifying the metabolites produced by CYP4Fs. It is sometimes difficult to distinguish the *ω*, *ω*-1, and *ω*-2 metabolites of CYP hydroxylases, but this can have physiological and pharmacological importance. For example, 19-HETE is produced by a number of CYPs and is an antagonist of 20-HETE [32]. We have provided experimental details for the identification of 18- and 19-hydroxy LTB_4_ [21, 22], but further studies are needed to clarify the roles of these metabolites. Another possibility is that CYP4F18 is redundant for LTB_4_ metabolism, because mouse neutrophils have an alternative pathway for LTB_4_ inactivation involving a 12-hydroxydehydrogenase that is not affected in* Cyp4f18* knockouts [22]. We speculated that CYP4F18 might have an alternative function in mouse neutrophils and that its homologue evolved to be the dominant LTB_4_-metabolizing enzyme in humans coincident with its ability to generate 20-hydroxy LTB_4_. This would predict that* Cyp4f18* knockout neutrophils exhibit LTB_4_-independent phenotypes.

Unexpectedly, a difference in response to complement component C5a was identified using the* in vitro* chemotaxis assay. Compared to wild-type neutrophils,* Cyp4f18* knockout neutrophils show an increase in chemotaxis of 2.3-fold at 10 ng/mL C5a and 1.9-fold at 100 ng/mL C5a (Figure 2). Primary chemoattractants such as C5a stimulate LTB_4_ synthesis and secretion, which amplifies neutrophil chemotaxis [20, 26]. Therefore, it is possible that increased C5a-dependent chemotaxis in* Cyp4f18* knockout neutrophils might be caused by increased levels of LTB_4_ or other eicosanoids arising from endogenous synthesis. We used two different inhibitors to investigate this possibility: a FLAP inhibitor (MK 886) that blocks activation of 5-lipoxygenase and a cPLA2*α* inhibitor that blocks eicosanoid production by preventing release of arachidonic acid from membrane phospholipids. These inhibitors reduced C5a-dependent chemotaxis to an equivalent degree in both wild-type and* Cyp4f18* knockout neutrophils, such that the relative difference in knockout cells compared to wild-type remained unchanged (Figure 2). A BLT1 antagonist increased C5a-dependent chemotaxis in both wild-type and* Cyp4f18* knockout neutrophils (Figure 2), possibly by blocking cross-desensitization between the chemoattractant receptors [30]. The relative difference in chemotaxis was undiminished following treatment with the BLT1 antagonist: 2.8-fold higher in* Cyp4f18* knockout neutrophils compared to wild-type (Figure 2). Overall, the data suggest that the increase in C5a-dependent chemotaxis in knockout neutrophils is unrelated to LTB_4_, other eicosanoids, or receptor cross-desensitization. An alternative possibility is that CYP4F18 metabolizes a novel lipid substrate involved in the downstream signaling pathways and relay systems that mediate C5a activity (C5a is a polypeptide and therefore not a CYP substrate).

CYPs typically have broad and overlapping substrate specificity, and redundancy of function is a consistent problem with CYP knockout studies. It is often difficult to observe a phenotype for deletion of one CYP* in vivo*, because of the effects of multiple related CYPs in complex tissue environments. There are advantages to studying* Cyp4f18* knockout neutrophils* in vitro*, because CYP4F18 is the only CYP4 transcript expressed at high levels in these cells (Figure 3). Another problem with CYP knockout studies is that deletion of one CYP sometimes leads to up- or downregulation of a different CYP that generates an observed phenotype. For example, knockout of* Cyp4a14* led to male-specific hypertension that was caused by upregulation of* Cyp4a12* [33]. We compared expression of all members of the CYP4 family in wild-type and* Cyp4f18* knockout neutrophils and detected no differences other than loss of* Cyp4f18* in the knockout (Figure 3).

We used a mouse model of DSS colitis for a preliminary investigation of the consequences of altered C5a chemotaxis* in vivo*. A C5a receptor antagonist ameliorates pathology in this model [31], and genetic association studies link variants of the* CYP4F2 and CYP4F3* genes with celiac disease in humans [9]. Mice were treated continuously with 4% DSS for 9 days, but the profile of weight loss, disease activity, and colonic tissue MPO was not significantly different in wild-type and* Cyp4f18* knockout mice (Figures 4 and 5). Selection and design of future* in vivo* strategies will benefit from more* in vitro* information about* Cyp4f18* knockout neutrophils. A comparison of lipid metabolism in wild-type and knockout neutrophils will help to determine if novel CYP4F18 substrates are relevant to C5a chemotaxis and might point to other activities affected by loss of CYP4F18.

## 5. Conclusions

CYP4F enzymes have the ability to catalyze oxidation of a diverse range of lipid substrates related to inflammation. Therefore, these enzymes are emerging as potentially prominent players in immune regulation. A significant challenge is to identify physiologically relevant substrates among multiple possibilities and to assign functions to individual CYPs. Knockout studies are problematic because of the ability of related CYPs to compensate for function or to change expression and lead to phenotypes that are unrelated to the deleted CYP. Neutrophils provide a useful tool for our studies of* Cyp4f18* knockout mice: CYP4F18 is the only CYP4 enzyme expressed at high levels in these cells, and other CYP4 enzymes do not change expression in* Cyp4f18* knockout neutrophils. In this report we demonstrate that there is no difference in LTB_4_-dependent chemotaxis of mouse neutrophils that lack CYP4F18, despite the high activity of CYP4F18 for LTB_4_ as a substrate. This is significant, because many studies assume that CYP4Fs regulate LTB_4_ function based on known activity as LTB_4_ hydroxylases. We identified an unexpected role for CYP4F18 in regulating C5a-dependent neutrophil chemotaxis, and this was independent of LTB_4_. Further studies of* Cyp4f18* knockout neutrophils* in vitro* will inform the design of* in vivo* strategies to investigate immune regulation.

## Figures and Tables

**Table 1:** Summary of primers.

TaqMan primers for real time PCR	
(Applied Biosystems/Life Technologies)	
C5ar1	Mm00500292_s1
Cyp4a10	Mm01188913_g1
Cyp4a12a	Mm00514494_m1
Cyp4a12b	Mm00655431_gH
Cyp4a14	Mm00484135_m1
Cyp4a29	Mm01188902_g1
Cyp4a30b	Mm01181463_m1
Cyp4a31	Mm03047753_m1
Cyp4b1	Mm01193710_m1
Cyp4x1	Mm01181487_m1
Cyp4f13	Mm00504576_m1
Cyp4f14	Mm00491623_m1
Cyp4f15	Mm00506542_m1
Cyp4f16	Mm00775893_m1
Cyp4f17	Mm01345625_m1
Cyp4f39	Mm00624134_m1
Cyp4f40	Mm01342246_m1

Custom TaqMan primers	

Cyp4f18	
Forward	AGTGGACTTTCCTGGATCCTGTAC
Reverse	GGCAGCGCTCCTGGTATTC
Probe	ACCTGGCAAGACACC
Cyp4f37	
Forward	TCCCGCCTCAGATGTTTCC
Reverse	CCCAAGTGACCCAAAAACCA
Probe	TCAGCCTCCTAAAAGA
Mouse blt1	
Forward	CCGACTTGGCTGTGTTGCT
Reverse	GTGCCTCGAGCCAGAAAGTG
Probe	ACTGCTCCCTTTTTC

Sequences are shown in 5′ to 3′ direction.
